# Mainstreaming orphan millets for advancing climate smart agriculture to secure nutrition and health

**DOI:** 10.3389/fpls.2022.902536

**Published:** 2022-08-12

**Authors:** Piyoosh K. Babele, Himabindu Kudapa, Yogeshwar Singh, Rajeev K. Varshney, Anil Kumar

**Affiliations:** ^1^College of Agriculture, Rani Lakshmi Bai Central Agricultural University, Jhansi, Uttar Pradesh, India; ^2^Centre of Excellence in Genomics and Systems Biology, International Crops Research Institute for the Semi-Arid Tropics, Hyderabad, India; ^3^Murdoch's Centre for Crop Research & Food Innovation, State Agricultural Biotechnology Centre, Murdoch University, Murdoch, WA, Australia

**Keywords:** COVID-19, drought, millets, multi-omics, nutritional security, policy-making

## Abstract

The ever-changing climate and the current COVID-19 pandemic compound the problems and seriously impact agriculture production, resulting in socio-economic insecurities and imposing health implications globally. Most of the poor and malnourished population in the developing countries depends on agriculture for food, income, and employment. Impact of climate change together with the COVID-19 outbreak revealed immense problems highlighting the importance of mainstreaming climate-resilient and low input crops with more contemporary agriculture practices. Orphan millets play a vital role in the poor and malnourished population's livelihood, food and nutrition security. Recognizing their unique potential, the United Nations-Food and Agriculture Organization has announced the year 2023 as the “International Year of Millets”. However, despite the unique properties for present and future agriculture of orphan millets, their cultivation is declining in many countries. As a result, millets have gained attention from researchers which eventually decelerated “multi-omics” resource generation. This review summarizes the benefits of millets and major barriers/ bottlenecks in their improvement. We also discuss the pre- and post-harvest technologies; policies required to introduce and establish millets in mainstream agriculture. To improve and ensure the livelihood of the poor/malnourished population, intensive efforts are urgently needed in advancing the research and development, implementing pre- and post-harvest technological intervention strategies, and making favorable policies for orphan crops to accomplish food and nutrition security. National and international collaborations are also indispensable to address the uncertain effects of climate change and COVID-19.

## Introduction

Agriculture is often considered a “national security” and is on the highest priority as agriculture products are necessary for survival and existence. Climate change and increasing human population has become a persistent issue causing immense distress globally (Fróna et al., 2019). Similar to climate change, a pandemic is also a global risk. The novel Coronavirus disease 2019 (COVID-19) pandemic has further disrupted many agriculture and supply chain activities, putting pressure on livelihoods for food and nutrition security. Combined natural and human disasters have negative impacts on agriculture and food system eventually threatening energy and nutritional demands of growing human population (Mishra et al., 2021; Rasul, 2021). Farmers are now facing two-fold challenges of climate change extremes and at the same time dealing with the uncertainty in production, marketing, transportation, and income arising from the COVID-19 pandemic (Bisoffi et al., 2021; Rasul, 2021). These incidences directly or indirectly impact the agriculture sector and food system, resulting in socio-economic insecurities and imposing health implications, particularly in marginalized populations (Bisoffi et al., 2021; Mishra et al., 2021). Tackling these challenges necessitates a paradigm shift from the existing incremental adaptation strategies toward transformative substitutes that emphasize human health, nutrition, and environmental sustainability. The current natural and human disasters make it even more imperative to shift toward a climate-resilient agriculture system (Bisoffi et al., 2021; Rasul, 2021).

One possible solution to tackle these issues is “orphan crops”, which can diversify crop production and provide more nutritious food sources (Ye and Fan, 2021). While more than 5,000 plant species are recorded as food plants, <20 species provide most of the world's food, and three major cereals, i.e., rice, wheat, and maize, account for ~60% of the calories and ~56% of the protein that humans consume directly from plants (Dawson et al., 2019). Many edible species of “orphan crops” are mostly ignored by farmers and cultivated in restricted regions, are non-commodity crops and not extensively commercialized, and have gained little attention from researchers and other development initiatives globally (Sood et al., 2016; Ribaut and Ragot, 2019; Tadele, 2019). Orphan crops constitute a major crop group, including cereals, legumes, fruit, and root crops, identified as underutilized, lost, minor, or neglected crops and as crops for the future especially in developing countries (Tadele, 2019). Orphan crops perform better than major crops under the changing climate scenario because they have unique features like diverse adaptation on marginal lands, minimal water requirement, adaptability/resistance to biotic and abiotic stresses, and nutritional superiority than major cereal staples (Nagaraja and Das, 2016; Varshney, 2021). These features make them an excellent choice to complement major staple foods for crop and dietary diversity. Of the orphan crops, millets are the world's ancient and most versatile grains as part of food culture in developing countries for many years (Bandyopadhyay et al., 2017; Varshney, 2021). Millets play a vital role in food security, nutrition, and income generation to resource-poor farmers in developing countries. Yet, despite its traditional importance, it has been marginalized in recent decades. Also, they got very little scientific attention in developing genetic and other “omics” resources and breeding for improving yield under stress conditions (Bandyopadhyay et al., 2017; Kumar et al., 2018).

While the current pandemic poses some serious challenges for the health and food system, it also offers an opportunity to accelerate revolutions in the health and agriculture sector to build its resilience in changing climate and post COVID-19 era (Fróna et al., 2019; Mishra et al., 2021; Rasul, 2021). The extraordinary challenges posed by the ever changing climate and COVID-19 outbreak requires very urgent and decisive policies to ensure food and nutrition security to save people's lives and livelihoods. Several multidirectional steps are required to warrant the quantity and quality of food needed to feed more than 7 billion people. Among them, a large number have already entered poverty as climate and disease shocks impacted their livelihoods (Fróna et al., 2019; Bisoffi et al., 2021). In the immediate run, governments must accomplish multiple demands responding to the health disaster, addressing the consequences of economic downfall, by ensuring the smooth functioning of agriculture (first priority) and other economic sectors (Bisoffi et al., 2021). For example, in India, the government has already increased its focus on nutrition (besides food) security and raising farmers' income (rather crop productivity). Changing consumer behavior with suitable programs and incentives is already on the government's agenda (Bisoffi et al., 2021). The aim of this review is to highlight the importance of millets in a sustainable agriculture system, and to address the current research advances in millet and future research ideas for further improvement and adoption. We summarize the unique properties of millets and briefly discussed about the adaptive mechanisms against drought stress thus emerging as a potential crop to understand biology of stress mitigation/adaptation. We discuss the nutritional and health benefits of millets for boosting immunity amidst post COVID-19 pandemic. We discuss the possible strategies such as creating policies, research projects for introducing millets in mainstream agriculture. Finally this article highlights the recent progresses and outcomes of multi-omic based research findings on millets. We recognize that systems biology integration with multi-omics datasets can enhance our understanding of molecular regulator networks for millet improvement. Moreover, millet specific agri-processing technologies are urgently required for value addition and to accelerate climate-resilient and nutrient-dense millets for sustainable agriculture, the environment, and healthy food systems.

## Benefits of establishing orphan millets in mainstream agriculture

### Millets for climate smart agriculture

**Figure 1:**
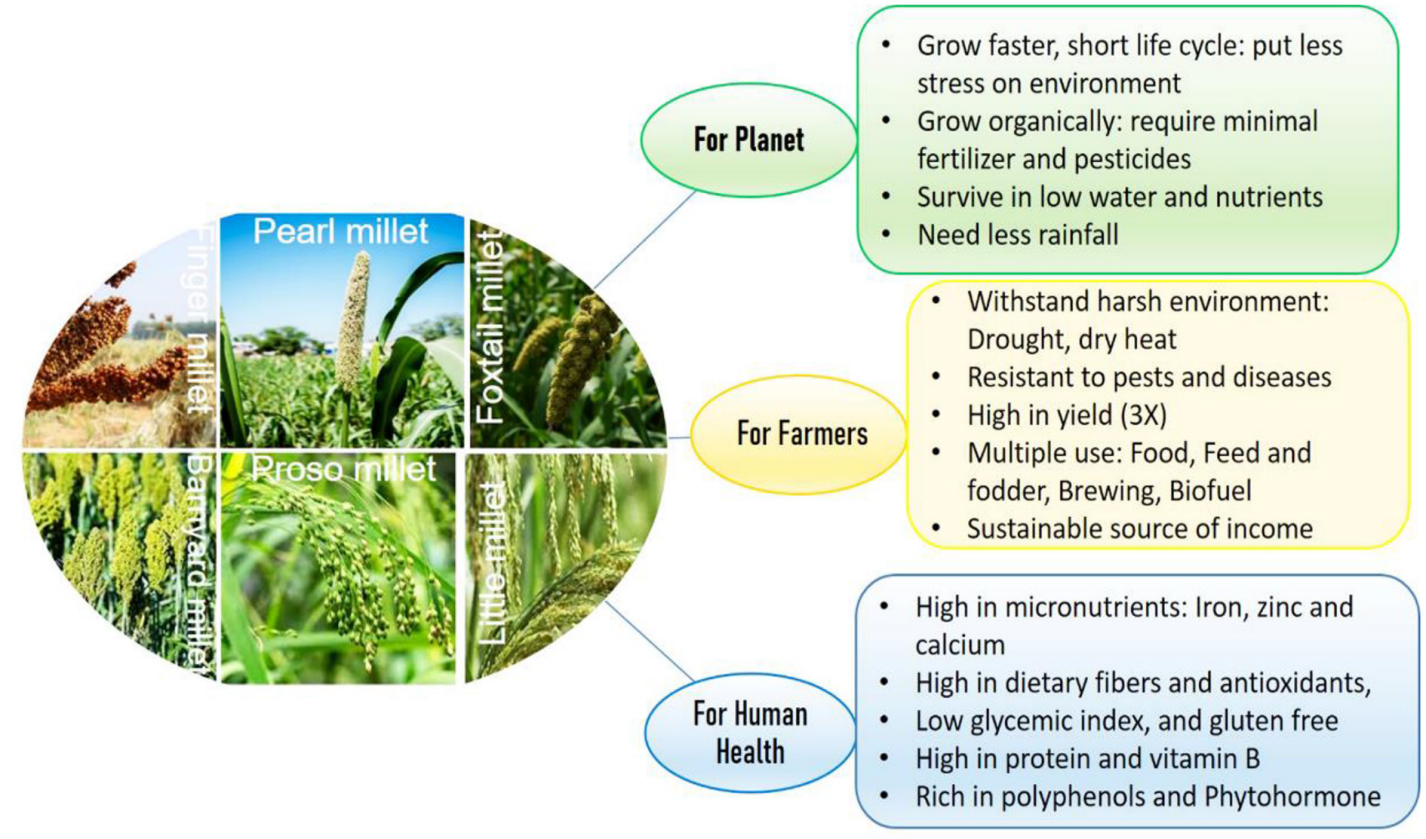
Unique properties of millets for climate smart agriculture, ensuring human health, food and nutritional security.

The demand for food will increase proportionally with a growing population. While maize, rice and wheat have been adopted as the major staple cereals, millets and other orphan crops are lagging behind. There is a lesser possibility of crop improvement (major staple cereals) production as the world is already facing the challenges of drylands expansion, soil degradation, and groundwater scarcity (Bisoffi et al., 2021). For example, in India, according to the National Rainfed Area Authority (NRAA 2020) report (https://www.nraa.gov.in/), even after utilizing maximum irrigation potential, about half of the total irrigated land will continue to remain unirrigated. These alarms are forcing us to promote alternatives to major cereal crops. Millets are the best choice among orphan crops and their cultivation can solve this problem as they can be grown on shallow, low fertile soils with a varied (ranging from 4.5 to 8.0) pH (Rathinapriya et al., 2020). There are many small millets such as finger, foxtail, proso, barnyard, Kodo, little, guinea, browntop, teff, fonio, and job's tears. Millets can be an easy replacement for wheat and rice. Further, millets like pearl and finger millet can grow up to a soil salinity of 11–12 dS/m, while rice has poor growth and productivity on a soil having salinity higher than 3 dS/m (Rathinapriya et al., 2020). They are considered as a poor man's crop due to their significant contributions to a resource-limited population diet offering several opportunities for their cultivation in developing countries (Figure 1).

### Drought and millets: Impact and adaptation

**Figure 2:**
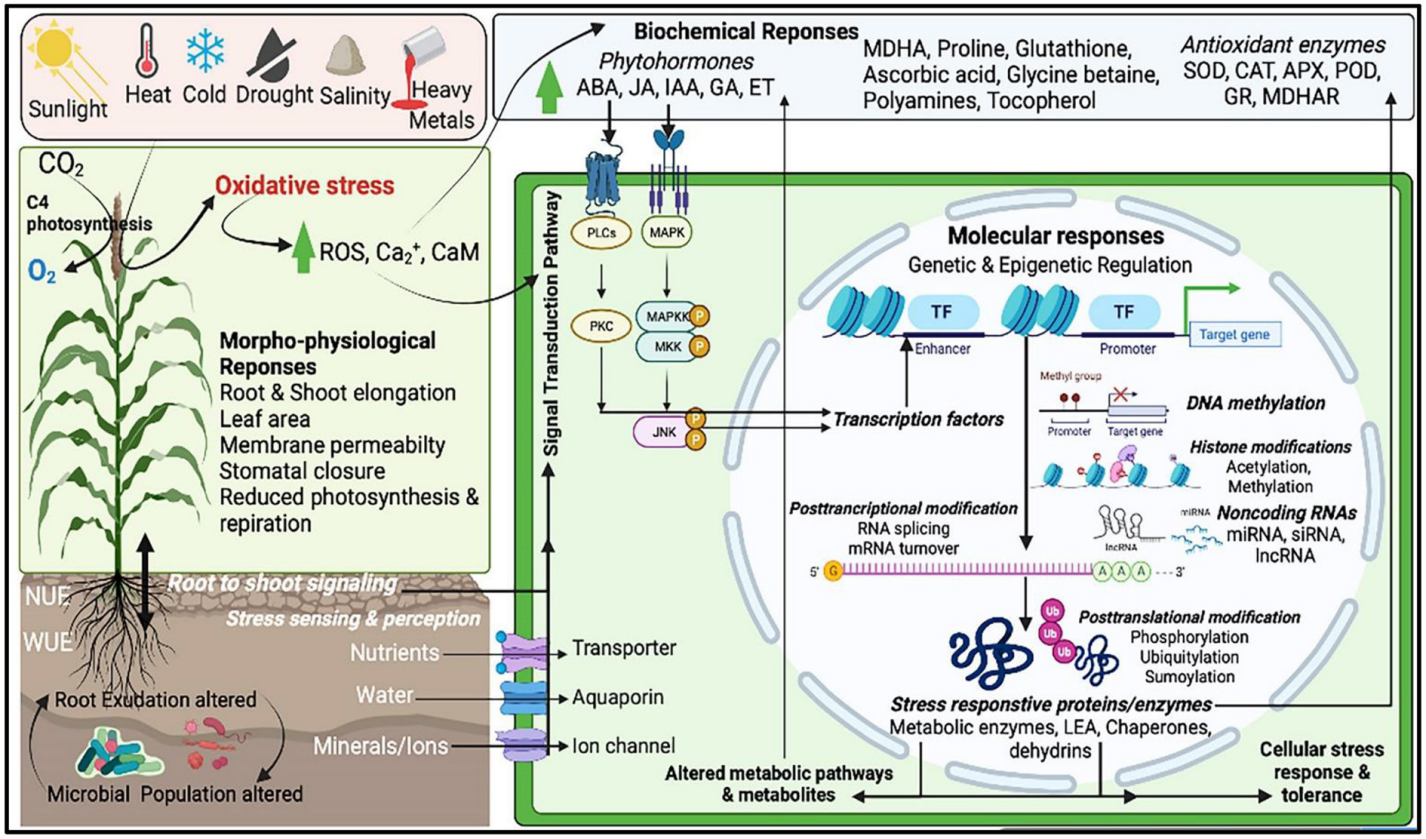
A schematic framework of abiotic stress response sensing, signaling and regulation in millet cells. Abiotic stresses alter morphophysiological, biochemical and molecular processes and also by modulating the rhizosphere properties and functions in millets. Millet cells can sense and perceive stress signals in several cellular compartments through signaling receptors and activate the signaling cascade through signal transducers (e.g., secondary messengers such as ${\text{Ca}}_{2}^{+}$ ROS, phytohormones, polyamines etc.). These signaling messengers trigger the molecular regulatory pathways involving both genetic and epigenetic factors such as post-transcription and post-transactional modifications *via* DNA methylation, histone modifications and non-coding RNAs activities. Stress signals activate the stress responsive transcription factors which then regulate the stress inducible gene and protein expression. These stress induced functional protein and enzymes have direct role in metabolism and stress adaptation and tolerance.

**Figure 3:**
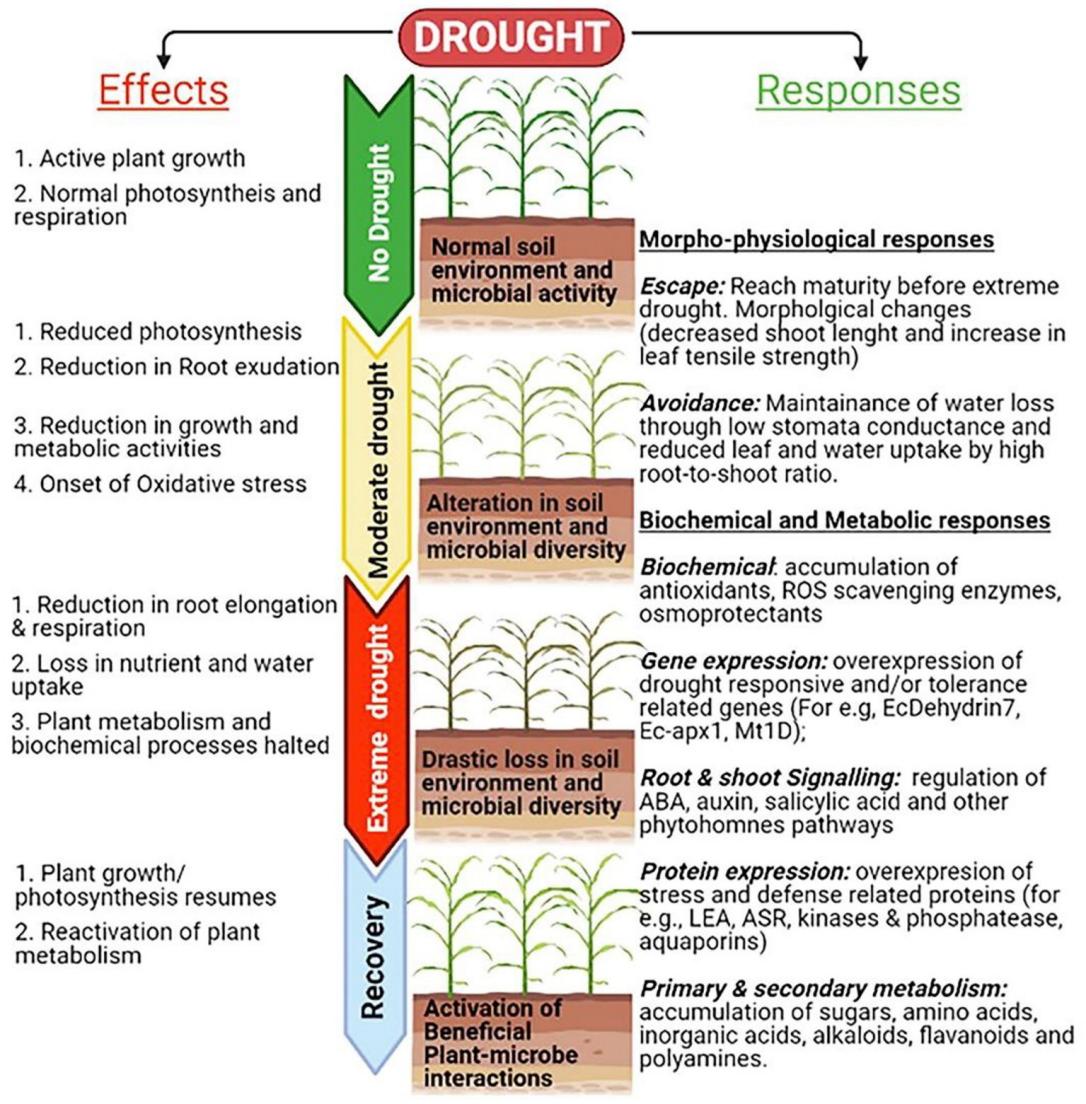
Drought responses and adaptive strategies linked to numerous morpho-physiological, molecular, and biochemical processes that confer better tolerance to environmental stresses in millets compared to major cereals.

Crop productivity is affected by a number of biotic and abiotic stresses. Rapid changes in climate led to major losses of arable land used for crop production and imposing abiotic stresses during critical plant growth and development stages causing yield losses. In semi-arid and arid regions, abiotic stress such as drought, extreme temperature (cold, frost and heat), flooding, salinity etc. are the major yield limiting stressors. Millets encompass numerous morpho-physiological, molecular, and biochemical properties that confer better tolerance to environmental stresses than major cereals. Molecular mechanisms underlying the plants responses to abiotic stresses include multitude of processes including sensing, signaling, transcription, transcript processing, translation and post-translational protein modifications which are governed by both genetic and epigenetic factors (Figure 2). Among all major abiotic stresses, increased drought and heat due to climate change adversely affect current crop production and alone cause more annual losses. The climate change models predict that drought stress would continue as a major abiotic limitation for food production (Simmons et al., 2020). Many reports show indirect associations between drought and the rise in malnutrition rates, poor health, hunger, starvation, food and water insecurity (Ye and Fan, 2021). Millets being naturally drought tolerant, stands as best alternative in the semi-arid and arid environments. Several studies on millets showed that drought impacts include growth, yield, membrane integrity, pigment, osmotic adjustment, water relations, and photosynthetic activity and also modulates the root-associated microbiome structure and activity (Gupta et al., 2017; De Vries et al., 2020; Simmons et al., 2020; Veach et al., 2020). Millets encompass numerous morphophysioloical, biochemical and molecular traits that confer superior adaptation to drought than major cereals (Figure 3). For example, the rainfall requirement of pearl and proso millet is 20 cm, which is many folds lower than rice as they require more than 120–140 cm (Kumar et al., 2018). The short life-cycle of millets (~10–12 weeks) as compared to other major crops (20–24 weeks) also support them in stress mitigation. Several traits such as short stature, small leaf area, thickened cell walls, and dense root system also contribute to circumventing the stress and long-term consequences (Bandyopadhyay et al., 2017). Also, they have a C4 (Hatch and Slack Pathway) photosynthetic system that is highly advantageous to survive in high temperatures and low moisture. In the C4 system, the light-dependent reactions (occur in mesophyll cells) and the Calvin cycle (in bundle-sheath cells) are physically separated. Thus, the C4 mechanism enhances the concentration of CO_2_ in bundle sheath, which overpowers photorespiration, and plants can keep their stomata closed during the daytime, thus avoiding water loss (Lundgren et al., 2014). Millets have enhanced photosynthetic rates at warm conditions and confer immediate water and nitrogen use efficiency, which is ~1.5- to 4-fold higher than C3 photosynthesis (Wang et al., 2012). For instance, *Setaria italica* requires just 257 g of water to produce dry biomass of 1 g, whereas maize and wheat require 470 and 510 g, respectively (Nadeem et al., 2020). Additionally, C4 photosynthesis provides secondary benefits to millets, including better growth and ecological performer in warm temperatures, enhanced flexible allocation patterns of biomass, and reduced hydraulic conductivity per unit leaf area (Lundgren et al., 2014). These examples offer opportunities for the promotion of these crops to a higher level of production in the changing climate scenario. Several studies have shown stress adaptation/mitigation strategies in millets. For example, pearl millet adjusts their flowering phenology according to rainfall pattern (Bidinger et al., 2007). Compared to maize, pearl millet can modulate their membrane dynamics better for water permeability to attain better water status during osmotic stress (Bandyopadhyay et al., 2017). An increase in leaf tensile strength and root length was reported in teff and little millet under drought (Balsamo et al., 2006). Several biochemical events, e.g., reactive oxygen species (ROS) regulation, enhances ROS scavenging enzymes (catalase and superoxide), and other stress-related proteins. The accumulation of antioxidants and osmolytes have been reported in response to abiotic stresses in millets (Ajithkumar and Panneerselvam, 2014). These qualities of millets make them a suitable model system holding the potential for research to explore the stress-responsive traits and delineate the mechanism of stress at the physiological, biochemical, and molecular levels. The information is crucial for the millet improvement.

### Millets for nutritional and health security during post COVID-19

The poor sections of society especially in developing countries are always the hardest hit in any disaster or pandemic situation. For example, nearly 90% of India's agricultural sector comprise of small and marginal farmers, are particularly vulnerable to economic shocks including COVID-19 distress. Boosting the immune system is the best defense to support the human body's natural ability to defend against pathogens (e.g., viruses, bacteria, and fungi). The immune system is actively combating pathogens, but its activity is enhanced if an individual got infected. This hyperactivity is accompanied by an increased rate of metabolism, requiring energy sources, substrates for biosynthesis, and regulatory molecules. Therefore, it is essential to fuel the immune system with the right quantity and quality of nutrients, which are all ultimately derived from the diet we normally consume. When people are exposed/infected to deadly coronavirus, the immune system is compromised and it is crucial to trigger the immune response back to normal by nutritious food intake (Calder, 2020). Those who recovered from COVID-19 must adhere to a healthy lifestyle by following a strict diet to boost immunity. Millets are more nutrition-rich than other major cereals in their calcium, iron, potassium, magnesium, and zinc content including other essential molecules such as vitamins, amino acids, and fatty acids (Muthamilarasan and Prasad, 2021). A summary of the nutritional profile of millets is shown in Table 1. They are a high source of antioxidants (quercetin, curcumin, ellagic acid, and other useful catechins), thus considered an immunity booster. Further, millets aids in detoxification by eliminating harmful toxins, free radicals and neutralizing enzymes in organs, eventually preventing various health issues such as cancer and heart disease. Many vitamins (A, B6, B12, C, D, E, and folate) and trace elements (zinc, copper, selenium, and iron) have been proven to have key roles in supporting the human immune system and reducing the risk of infections. Therefore, it would seem prudent for individuals to consume sufficient amounts of essential nutrients to support their immune system to help them cope with pathogens. The gut microbiota also plays a crucial role in maintaining and regulating the immune system. Gut dysbiosis is a feature of disease including many infectious diseases and has been described in COVID-19. Dietary styles to accomplish a healthy human microbiota can also benefit the immune system (Peterson et al., 2010; Rodríguez et al., 2020).

**Table 1:** Nutritional values of millets (per 100 g edible portion, dry weight basis).

**Nutrients**	**Pearl**	**Finger**	**Foxtail**	**Little**	**Proso**	**Kodo**	**Barnyard**
Carbohydrates (g)	67.5	72.6	60.9	67	70.4	65.9	67
Starch (%)	60.5	59.0	59.1	60.3	56.1	72.0	60.3
Protein (%)	14.5	7.3	11.7	7.7	11	8.3	6.2
Fat (%)	5.1	1.3	3.9	4.7	3.5	1.4	4.8
Crude fiber (%)	2	3.6	7	7.6	9.0	9.0	13.6
Total dietary fiber/100 g	7.0	19.1	19.11	-	8.5	37.8	13
Total phenol (mg/100 g)	51.4	102	106	21.2	0.10	368	26.7
Ash (%)	2	3	3	6.9	3.6	3.6	4
Energy (kcal)	336	361	331	341	341	309	307
**Minerals and vitamins (mg/100 g)**.							
Ca	42	344	31	17	8	35	22
P	240	283	290	220	206	188	280
Fe	11	3.9	2.8	9.3	2.9	1.7	18.6
Mg	137	137	143	61	114	110	82
Na	10	11	1.3	7.9	5	4.8	-
K	390	408	364	126	195	141	-
Cu	1.06	0.47	0.59	0.05	0.8	1.60	0.60
Mn	1.15	5.49	1.16	0.68	1.6	1.10	0.96
Zn	3.1	2.3	3.51	3.7	1.7	0.70	3
Thiamine	0.38	0.42	0.59	0.30	0.41	0.15	0.33
Riboflavin	0.21	0.19	0.11	0.09	0.28	0.09	0.10
Niacin	2.8	1.1	3.2	3.2	4.5	2	4.2

Sources: Chandra et al. (2016), Kumar et al. (2016), and www.millets.res.in.

Being rich in magnesium and potassium, millet reduces blood pressure by acting as a vasodilator thus minimizes the risk of heart attacks or strokes, particularly in atherosclerosis; optimizing the circulatory system is one of the best ways to protect cardiovascular health. Millets are rich in lignans, upon digestion they can be converted into animal lignans by gut microbiota, and those animal lignans have been shown to protect against certain chronic diseases, like cancer and heart disease (Peterson et al., 2010). Millets constitutes a range of health-promoting nutrients, such as proteins, dietary fibers and phenols. High dietary fibers and phenolic content as well as low glycemic index make them advantageous for diabetic patients. Consuming millet products can decrease fasting glucose by 32% and remove insulin resistance by 43% (Muthamilarasan and Prasad, 2021). Magnesium is also considered important for increasing the activity of insulin and glucose receptors in the body, thereby controlling diabetes. A 30% reduction in diabetes has been reported in populations segregated between diets with or without magnesium (Carlson et al., 2018). High levels of dietary fiber also make them ideal for lowering cholesterol by eliminating toxic “bad cholesterol” (LDL) from the system while supporting the effects of “good cholesterol” (HDL). High fibers and minerals in millet can help in eliminating problems like constipation, excess gas, bloating, and cramping (Rodríguez et al., 2020). Regulation in the gastrointestinal system improves nutrient retention and also help optimize the functioning of kidney, liver, as these organs are directly related to the body's metabolic activities and ultimately part for the immune system (Annor et al., 2017). Healthy digestive system reduces the chance of more serious gastrointestinal problems like gastric ulcers or colon cancer. Recent research has documented that chances of breast cancer can be reduced by more than 50% through intake of dietary fiber rich diet (Carlson et al., 2018). A positive outcome of the COVID-19 could be described as a change in our eating habits with a balanced/nutrient rich diet and this helped millets attain global attention due to their nutraceutical properties.

## Strategies for introducing millets in mainstream agriculture

**Figure 4:**
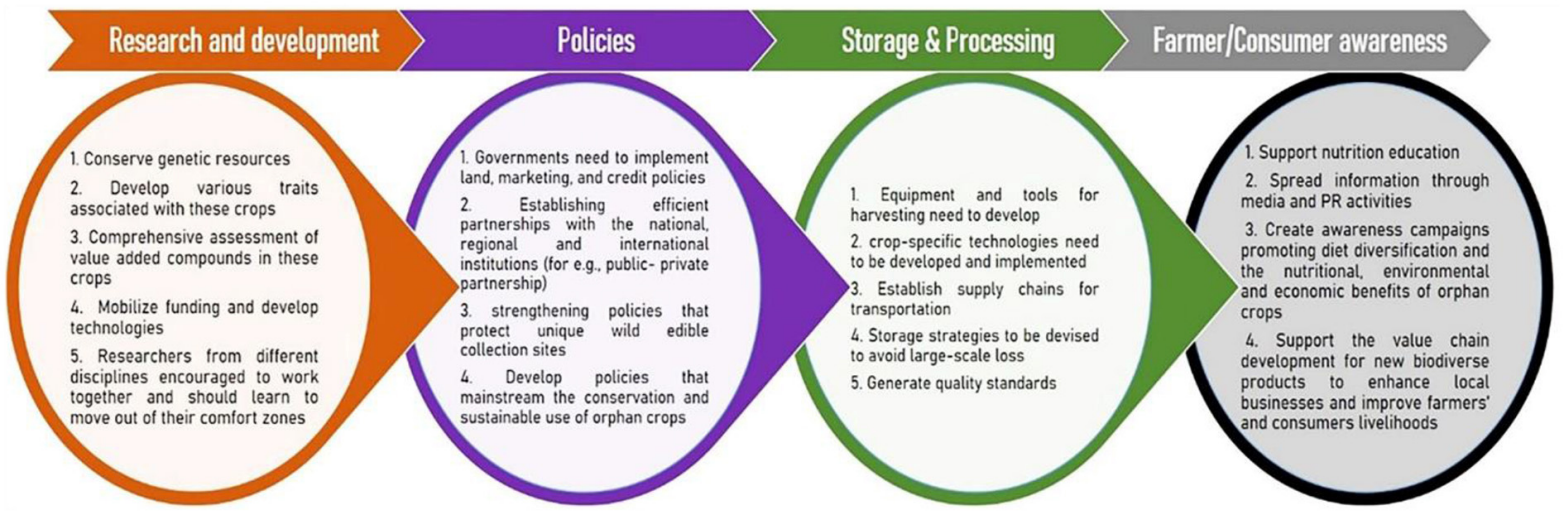
Multi-stakeholders' strategies and coordinated approaches at the local, regional, and international levels for the promotion of orphan crops for sustainable agriculture development.

There are several common challenges in tackling climate change and pandemics. Both calamities feature great ambiguity in their inception, extent, and degree of severity. When experienced together, these uncertainties and disruptions are compounded, which may lead to severe economic imbalance and nutritional insecurity. The current pandemic and climatic disasters have important lessons for climate change policy and agricultural sustainability (Mishra et al., 2021). Scientists have developed the capacity to better predict and initiate early warnings about natural disasters and pandemics. While science does not have all the answers, governments that rely on scientific advice are likely to better manage human and natural disasters. To respond to crises like these, science needs to assist in designing risk governance strategies, namely institutions, rules and capabilities that balance central and regional responsibilities to achieve socially improved outcomes (Priyadarshini and Abhilash, 2021). Hopefully, these crises will lead to further reliance on science in addressing phenomena such as climate change. In this section we outline few strategies and policy responses to better manage combined human and natural disasters in the future. To maximize the potential of orphan crops, coordinated approaches on the local, regional, and international levels have to be implemented, which required a multi-stakeholder system (Figure 4). These efforts are to be implemented under the aegis of the global sustainable development goals. However, large-scale production cannot be achieved without stakeholders' concerted efforts across the value chain, from research to production to marketing to end-use. Policy-making steps are critical in filling knowledge gaps and fostering connections between stakeholders in different sectors to promote greater awareness, production, marketing, integration of orphan crops and wild edible species in public programs to mainstream them in current agriculture. Growing interest among producer and consumer about climate-resilient features and health healing properties of millets underline the necessity of directing more research and development toward these crops. Now policies must favor the orphan or underutilized crops instead of tending the big staple crops (maize, rice, and wheat). In the post COVID-19 scenario, nutritional security must promote the underutilized crops for a food systems transformation (Priyadarshini and Abhilash, 2021).

### Creating policies and markets for millets

Although marketing is the most sustainable way to promote underutilized crops, establishing markets for orphan crops is not that simple. Favorable policies and a supportive environment by establishing an efficient partnership with the national, regional, and international institutions are important for improving and promoting orphan crops. Government should develop coordinated, multi-sectoral policies that recognize the importance of orphan crops and wild edible species. Also, public-private partnership (PPP) is considered an effective mechanism to bring together the public and private sectors to enhance agricultural sustainability in the developing world. Farmers and end-users should encourage to adopt and accept improved agriculture technologies for success in agricultural development. One way is to involve farmers in decision-making to recognize the potential of new technologies' when they are at an initial stage of development. A strong extension system must be established to connect the research and farming community (Borelli et al., 2020; Bisoffi et al., 2021). Some short-term support can be replacing fuel-propelled water engines with solar energy-driven irrigation systems, chemical fertilizers, and organic fertilizers, which can alleviate the coronavirus crisis and support climate mitigation (Mishra et al., 2021; Rasul, 2021). To obviate the current/post COVID-19 issues, policies must facilitate easy availability of machinery through government and private entities, farmer producer organizations (FPOs), or custom hiring centers with suitable incentives (Bisoffi et al., 2021; Rasul, 2021).

Governments need to form a conducive policy to build institutional capacity and implement land, marketing, and credit policies to encourage and support agricultural development. Implementation of the right type and locally applicable technologies need to be developed. Policies that promote and strengthen mainstreaming, conservation, and sustainable use of orphan crops should be formed. Policies that support public procurement mechanisms favoring the supply of orphan crops from farmers/collectors and those that support the value chain development for new biodiverse products to enhance local businesses and improve farmers' and collectors' livelihoods should be promoted. Government should support nutrition education using culturally and socially appropriate nutrition messaging, create awareness campaigns about the nutritional, environmental, and economic benefits of millets, and promote diet diversification programs (Borelli et al., 2020).

Furthermore, post-harvest processing and storage are limitations in the large-scale production of seed grains. For major cereals, organized processes and equipment are available to prevent post-harvest wastage; however, such machinery/technologies for harvesting and/or threshing are unavailable for all orphan crops. Considering all millet grains' unique morphologies and architectures, crop-specific equipment needs to be developed and implemented. Optimum temperature and humidity are required to prevent sprouting or rotting of millet grains. During long-term storage of millet grains beyond optimum conditions, they tend to become rancid due to their higher lipid content. Therefore, proper storage strategies are to be devised and these storage facilities would prevent large-scale loss of valuable produce. Large storage capacities serve as a reserve to rely on during natural calamities and avoid global hunger and malnutrition indices. Supply chains should also improve to ensure the transportation of the agricultural outputs that would benefit the farmers and stakeholders involved in cultivating orphan crops (Baldermann et al., 2016; Muthamilarasan and Prasad, 2021).

### Investment in research and development for orphan millet crops

It is important to mobilize research networks within academic and research institutions to understand orphan crops' agricultural and economic value. Also, the focus on invasive species research in developing countries needs to be prioritized. Stakeholders involved in agricultural research and development need to invest in agricultural innovation to improve production, marketing, or distribution. The germplasm of under-studied orphan crops should be properly collected and managed by researchers to avoid genetic material loss due to human and natural disasters (Ye and Fan, 2021). The germplasm needs to be made available to researchers from developed and developing countries to utilize the landraces' genetic diversity. Since biotic and abiotic stresses affect these crop's productivity, future research should also focus on exploring resistance or tolerance mechanism(s) against these environmental calamities (Baldermann et al., 2016; Muthamilarasan and Prasad, 2021). It is also important for researchers to equip each other with the appropriate skills for combating invasive species using new and holistic approaches. One of the ways to achieve this is to establish interdisciplinary collaboration and knowledge exchange. National and international funding agencies should also release funds for orphan crop research to map genes and their functions in more detail (Baldermann et al., 2016). Researchers should create partnerships with governmental and non-governmental organizations to conduct participatory research, wherein farming communities/farmers can contribute and exchange their knowledge with the scientific community considering the important role of indigenous knowledge in orphan crops.

## Advances in “multi-omics” resources for mainstreaming orphan millet crops

Orphan crops have characteristic features that can help promote these crops to a higher level of production. Plant biologists believed that increased production of orphan millets by improved breeding practices could reduce food security and malnutrition (Dawson et al., 2019; Muthamilarasan and Prasad, 2021). The “omics” resources for orphan crops lag compared to other cereal crops resulting in their slow improvements. Genetics, a primary input for breeding, and genomics, an efficient tool for their characterization, are imperative for improving crop species. Although the genomic resources information about molecular markers and physical/genetic maps for orphan crops, including millets, is increasing, these efforts are inadequate, which hampers their improvement (Varshney et al., 2012). A few partial genetic maps for millet species were constructed utilizing Restriction Fragment Length Polymorphism (RFLP), Amplified Fragment Length Polymorphism (AFLP), and Single-Strand Conformation Polymorphic (SSCP) expressed sequenced tags (ESTs) (Dida et al., 2007). Recent advances in next-generation sequencing (NGS) and the availability of whole-genome sequence (WGS) helps us to understand the genetics and mapping of quantitative trait loci (QTLs) for various traits. In addition to the genome sequence of pearl millet (Varshney et al., 2017), the most widely grown millet, genomes of five small millets, namely foxtail millet, finger millet, proso millet, teff, and Japanese barnyard millet, have been made available (Antony Ceasar et al., 2018). Of these small millets the genome of foxtail millet is the smallest (423–510 Mb) while finger millet has the largest one (1.5 Gb). Several alleles, QTLs, and novel genes have been identified in millets whose functional characterization has revealed their roles in conferring stress tolerance, nutritional and agronomic traits and summarized elsewhere (Bandyopadhyay et al., 2017; Antony Ceasar et al., 2018). Several whole-genome scan studies, e.g., genome-wide association studies (GWAS) and genomic selection (GS), facilitated by genotyping-by-sequencing (GBS), RAD-sequencing, and whole-genome re-sequencing (WGRS), were also performed in millets. Several stresses and nutritional, grain quality, related single nucleotide polymorphism (SNP), and simple sequence repeat (SSR) markers were identified (Gimode et al., 2016; Hittalmani et al., 2017; Sharma et al., 2018; Pujar et al., 2020). Regardless of this progress, knowledge on the genetic determinants of stress tolerance and the molecular machinery underlying stress tolerance is largely unknown in millets. In this context, extensive phenotypic screening to observe the natural genetic variations in stress tolerance across diverse millet germplasms is greatly needed. Altogether, it was anticipated that WGS based genomic improvements on millets drive researchers to perform high-throughput studies. The knowledge gained through these studies might explore our understanding of the roles of genes involved in nutrient signaling and abiotic stress responses and could be tapped for plant breeders to develop improved millet varieties (Raza et al., 2021).

Genomics has made significant progress in studying millets. Other omics approaches such as transcriptomics, proteomics, metabolomics, fluxomics and ionomics etc. are relatively new, and only a few reports are available on these crops. Rapid development and user-friendly high-throughput sequencing technologies have significantly improved knowledge about the relationship between genotype and phenotype. Transcriptomics, a functional genomics approach has been widely used in plant genetics research, revealing differentially expressed genes in numerous biological processes under certain environments. RNA-seq based transcriptome analysis is rapid, provides comprehensive information about gene expression, and characterizes the transcription factors and regulators to control various biological processes (Shivhare et al., 2020; Sun et al., 2020). In this aspect, foxtail millet was the first millet whose transcriptome was released by sequencing the total RNA of root, stem, leaf, and spike. After that, several transcriptome analysis studies on millets have been conducted under specific conditions (Sun et al., 2020; Wang et al., 2020).

As abundance and expression of RNAs (transcriptome) do not directly correlate with the protein and metabolite abundance, proteome and metabolome profiling would facilitate understanding of the functional aspects and cellular metabolism (Babele and Young, 2019; Babele et al., 2019). Further, a systems biology approach involving integration of multiple omics data, modeling, and prediction of the cellular functions is required to understand the flow of biological information underlying complex traits (Pazhamala et al., 2021). Proteomics can easily explore complex cellular biochemistry by understanding the structure and function of proteins under defined conditions. Proteins serve as links between the transcriptomic and metabolomic profile, thus dissecting cells' actual physiological and metabolic state (Babele et al., 2019). Furthermore, in stressful conditions, long-term cellular adaptation is governed by the synthesis of “stress-responsive proteins”. Detailed investigation of these proteins may delineate the mechanism of cellular adaptation under stress conditions. Metabolomics is the complete profiling of the metabolome defined as the complete set of small biomolecules or intermediates of cellular metabolism in a biological sample. The metabolome is determined by the coding capacity of the genome of a cell. It is mainly influenced by changes in the transcriptome and proteome but can also be affected by the chemical, physical, and nutritional environment of a cell (Babele and Young, 2019; Bueno and Lopes, 2020). While metabolomics can provide direct information on the complete set of metabolites, fluxomics describes the various methods that seek to determine metabolic flux, that is, the rates of *in vivo* metabolic reactions (Babele and Young, 2019). Both metabolomics and fluxomics are most closely related to the physiology and phenotype of an organism, organ, or cell, and they complement other omics technologies at the metabolite level (Babele and Young, 2019; Koley et al., 2019; Shih and Morgan, 2020). Identification of de-regulated proteins and metabolites using high throughput tools such as mass spectrometry and nuclear magnetic resonance (NMR) has provided new insights into proteomics and metabolomics (Babele and Young, 2019; Babele et al., 2019). We believe that the identification and characterization of de-regulated proteins and metabolites under certain stress environments provide necessary information regarding the cellular response at the level of their functions and, therefore, advance our understanding of the cell signaling and stress response pathways activated under stress conditions.

Millets are well adapted for low-input agriculture and display vast genetic variability for key mineral elements like, iron, zinc, and calcium and have better climate resilience as compared to major cereal crops. The functioning of stress-resilient and nutritional superiority traits is primarily dependent on the availability and accumulation of nutrients (minerals/ions) (Ceasar, 2019). Membrane transport proteins, including nutrient transporters, ion channels and aquaporins, play essential roles in the uptake of nutrients and water from the soil and in their radial transport to the root vasculature (Zelazny and Vert, 2014). Recently, both essential and non-essential minerals have been identified as key players in plant stress biology due to their multifaceted functions, thereby creating emerging field of research (Ali et al., 2021). Mineral nutrients, including Ca, S, Mg, Zn, Fe, Se, and Si, have also been displayed to mitigate the an array of abiotic stresses including drought, salinity, and heavy metal toxicity by boosting antioxidant activity, proline content, photosynthetic rate, and competing with heavy metal uptake, thus diminishing their response on cellular processes (Ali et al., 2021). Plants cells modulate their nutrient uptake to adjust growth and development in their ever-changing environment by relocalization of membrane transporters and ion channels in response to environmental cues therefore allow them to cope with the stresses (Zelazny and Vert, 2014; Pottosin et al., 2021). Therefore a better understanding on nutrient mobilization is critical for improving nutrient use efficiency and biofortification in millets and other cereal crops. Improving nutrient use efficiency of crops is important because marginalized farmers cannot afford to buy expensive fertilizers and their production is energy intensive and detrimental to the environment. Moreover, phosphate rock used for the production of fertilizers are non-renewable and depleting quickly. In this regard, ionomics (study of cellular metal, non-metal, and metalloid compositions), can provide new omics tools for identifying genes/proteins and their regulatory pathways related to mineral accumulation, transportation, cross-talk, and stress resilience (Huang and Salt, 2016; Ali et al., 2021). Ionomics will provide new platforms for not only understanding the function of the plant ionome during stresses will enable the development of biomarkers that can assess whether a plant has attained a certain biochemical or physiological state under different stresses and environmental conditions (Huang and Salt, 2016; Ali et al., 2021). Development of high-throughput elemental analysis technologies such as inductively coupled plasma-atomic emission spectroscopy (ICP-AES) and ICP-mass spectrometry (ICP-MS) has enabled the estimation of mineral contents (Huang and Salt, 2016). Earlier we have reviewed the molecular approaches for calcium biofortification and provided the information on molecular markers (transporters) for Ca uptake and accumulation in finger millet (Sharma et al., 2017). In a recent study authors identified various mineral nutrient transporter proteins (nitrogen, ammonia, phosphorous, sulfur, potassium, and micronutrients) in finger millet by bioinformatics tools (Maharajan et al., 2022).

A comparative transcriptome analysis under drought stress at two different developmental stages revealed up-regulation of several metabolic pathways such as flavonoid pathway, lignin biosynthesis, phenylpropanoid pathway, pigment biosynthesis, and other secondary metabolite pathways in drought-stressed pearl millet (Shivhare et al., 2020). In another study, 63,090 and 26,299 novel transcripts and genes, respectively, were identified which provided a clear understanding on drought tolerance mechanism in pearl millet. Among those, 6,484 genes, including 315 transcription factors and 128 transcription regulators, were differentially expressed under drought conditions (Sun et al., 2020). Recently a comparative physiological and proteomic analysis of drought stress responses in pearl millet demonstrated large differences in root morphology and photosynthetic machinery, revealing a stay-green phenotype (Ghatak et al., 2021). Subsequent tissue-specific proteome analysis of leaves, roots, and seeds led to identification of 12,558 proteins under well-watered and water-stress conditions. Proteome signatures related to root morphology and seed yield demonstrated the unexpected intra- and inter- species specific biochemical plasticity for stress adaptation in pearl millet. These quantitative proteomics data provide tissue- and phenotype- specific marker proteins of stress defense mechanisms for potential use in marker-assisted breeding (Ghatak et al., 2021).

In the case of foxtail millets, a comparative and quantitative proteomic investigation identified 2,474 differentially expressed proteins under drought stress conditions. Among these, 321 proteins were significantly expressed (252 up- and 69 down- regulated) and categorized as stress and defense responsive. Further, proteins/ enzymes involved in photosynthesis and carbon metabolism, ROS scavenging, protein synthesis and regulation, fatty acid and amino acid metabolism, polyamine biosynthesis, hormone metabolism, and cell wall modifications were also identified (Pan et al., 2018). Li et al. (2018) investigated comprehensive profiling, natural variation, and species-specific accumulation of primary and secondary metabolites using the LC-MS-based target metabolome profiling in foxtail millet. They identified and quantified more than 300 metabolites and classified them into flavonoids, phenolamides, hydrocinnamoyl derivatives, vitamins, and lysophosphatidylcholines. Results revealed these metabolites' roles in developmentally controlled accumulation and natural variation in different tissues/varieties.

Crossbreeding, mutation breeding, and transgenic breeding are currently the main methodologies for crop improvement in modern agriculture. Their stochastic nature constrains these procedures, generating and screening large numbers of mutants are time- and labor -intensive. Therefore, these untargeted breeding programs cannot keep pace with increased crop production demands (Muthamilarasan and Prasad, 2021). During the last decade, advances in genome editing (improving a trait by precisely modifying the target genes or regulatory elements or rearranging chromosomes in selected varieties) have revolutionized crop improvement programs (Hua et al., 2019). Among many genome editing techniques in plants (e.g., ZFNs and TALENs), clustered regularly interspaced short palindromic repeats (CRISPR)/CRISPR-associated protein 9 (CRISPR/Cas9) in particular, has emerged as a most powerful genome-editing tool enabling highly efficient and precise gene editing down to the level of a single base (Chen et al., 2019; Khan et al., 2019). Despite the technological developments being testified in CRISPR/Cas9 system for crop improvement, not much progress has been made toward applying these tools to improve orphan crops. This is primarily due to a dearth of data about the genes/proteins and complex metabolic pathways associated with particular stress conditions. Genome editing is proven as an effective tool for orphan crops. Lemmon et al. (2018) developed genomic resources and efficient transformation in the orphan *Solanaceae* crop “groundcherry” (*Physalis pruinosa*). CRISPR/Cas9 was used to mutate orthologs of tomato domestication and improvement genes controlling plant architecture, flower production, and fruit size, thereby improving these major productivity traits (Lemmon et al., 2018). However, accelerated crop improvement programs using these genome editing techniques and speed breeding are not currently practiced in millets. Exploiting such next-generation genomic approaches in orphan crops, including millets, should be promoted to minimize breeding time and facilitate the early release of resilient varieties.

**Figure 5:**
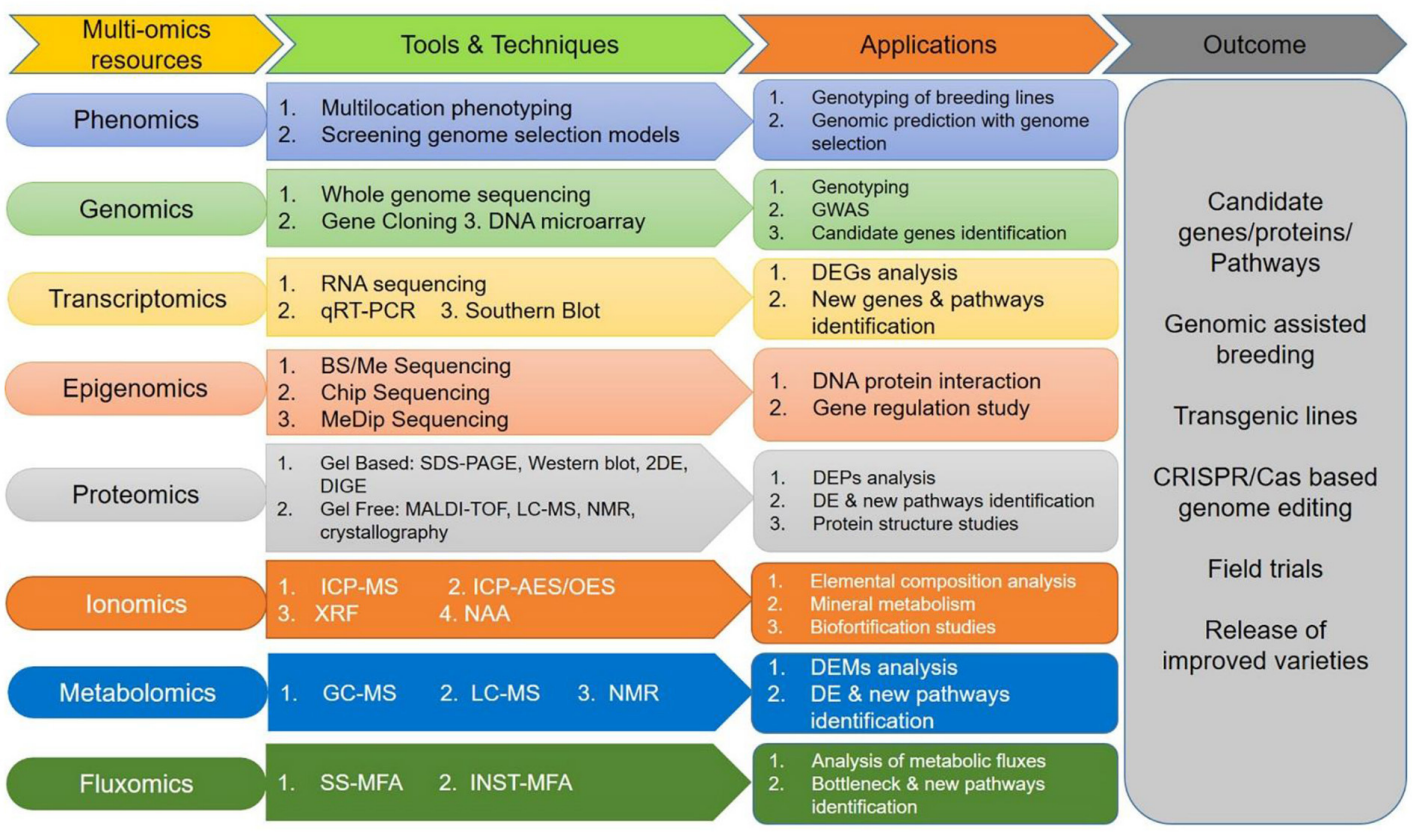
A “multi-omics” strategy for genetic improvement for mainstreaming millets for climate smart agriculture. GWAS, genome-wide association studies; BS/Me-Seq, bisulfite/methyl-sequencing; ChIP-Seq, chromatin immunoprecipitation and MeDip; Seq, Methylated DNA immunoprecipitation sequencing; qRT-PCR, Real-time quantitative reverse transcription polymerase chain reaction; 2DE, two-dimensional gel electrophoresis; DIGE, two-dimensional difference gel electrophoresis; MALDI-TOF, matrix-assisted laser desorption/ionization-time of flight; ICP-MS, inductively coupled plasma-mass spectrometry; ICP-AES/OES, inductively coupled plasma-atom/optical emission spectrometry; XRF, X-ray fluorescence; NAA, neutron activation analysis; GC-MS, gas chromatography-mass spectrometry; LC-MS, liquid chromatography-mass spectrometry; NMR, nuclear magnetic resonance; DEG, differentially expressed genes; DE, deregulated; DEPs, deregulated proteins; DEMs, deregulated metabolites; SS-MFA, steady-state metabolic flux analysis; INST-MFA, Non-stationary metabolic flux analysis.

Research and innovation are fundamental in every walk of life, including agriculture systems. Research on genetic analysis should focus on the frugality/rusticity of orphan crops instead of model plants and major crops. Recent advances in phenotyping and “omics” technologies, together with available germplasm diversity, should be utilized in millets improvement (Muthamilarasan and Prasad, 2021). We hypothesized that availability of more genomic resources advances our understanding of orphan crops' genetics. Also, to fully harness the underlying genetic potential and understand the molecular mechanism of abiotic stress tolerance, “multi-omics” approaches (Figure 5) to accelerate crop improvements in millets and other orphan crops are urgently needed. Integration of data gained from integrated omics would certainly explore candidate “genes/proteins/pathways” that could be manipulated using either transgene-based (overexpression/silencing) or genome-editing (CRISPR-Cas). It is hoped that genome editing tools will significantly contribute toward raising novel plant types with tolerance to multiple abiotic stresses and aid in public acceptance of these products in the near future.

## Conclusion and perspectives

Climate change has already had obvious effects on the environment and put challenges for the agricultural sector globally. Similar to climate change, the COVID-19 outbreak is also a global issue. These extreme climatic events (temperatures rise, rainfall variation, drought etc.) add to pressures on agricultural and food systems. This outbreak has disrupted many agriculture and supply chain activities, compounding food and nutrition security challenges, and sustaining livelihoods. Their impacts have the most negative effects on developing countries by adding resource problems, such as water scarcity, pollution, and soil degradation. There is huge genetic resources in terms of landraces, varieties are available in different millets but not much attention has been given to work the responsiveness of varieties under different soil types. Its an important area where epigenetic influences of genotype in response to different soil types interaction can be studied. Therefore, we advocate using the term “climate smart/resilient” agriculture considering several elements such as: (i) more prominent role for locally produced important minor (orphan) crops, (ii) increase in production of nutritious food and value addition to help the human immune system, (iii) creation of low-cost farming systems with less dependence on water and chemicals, (iv) leveraging agro-ecology to synthesize the environment with farming, and (v) nudging consumers' demand toward climate-resistant grains, and making farming more feasible for marginal farmers.

Orphan crops such as millets can contribute to sustainable food systems under climate change. Their resilient nature and outstanding potential to survive under low water availability and stressful environments serves as best alternative to staple cereal crops. Therefore, there is an argument to promote them to sustainably address challenges such as increasing drought and heat, food and nutrition insecurity, environmental degradation, and employment creation amidst COVID-19 in developing and poor countries. We need to tailor orphan crops according to the demands of each local environment by building research capacity. To date, limited time and resources are invested in research on them; that is why they are called “orphan crops”. Millet is a classic example of orphan crops with unique properties and potential for creating climate-smart agriculture. For more than 10 years, Biodiversity International has been working to conserve these species' genetic diversity, focusing on finding marketing avenues for production. We believe that the existing omics tools and methodologies can modernize the breeding of orphan crops; practical implementation of these modern omics technologies is needed. More funds and research programs are urgently required for the improvement of orphan crops. The success of efforts to develop rural economies to ensure food and nutrition security and poverty eradication depend on creating supportive agriculture policies, building climate change resilience in agricultural systems and innovations in farming practices managed by smallholders, and the widespread adoption of science and technology.

## Data availability statement

The original contributions presented in the study are included in the article/supplementary material, further inquiries can be directed to the corresponding authors.

## Author contributions

PB conceptualized the idea and wrote the original manuscript. PB, HK, RKV, and AK revised and finalized the manuscript. All authors read and approved the final manuscript.

## Funding

This work was supported by Ramanujan fellowship and Research Grant (File No. RJF/2020/000043) by Science and Engineering Research Board, Department of Science and Technology (SERB-DST), Government of India, New Delhi, India. RKV is thankful to Bill & Melinda Gates Foundation, USA (Grant ID# OPP1052922) for the support and SERB-DST for providing the J C Bose National Fellowship (SB/S9/Z-13/2019). HK acknowledges the SERB-DST (CRG/2019/005966) for the support.

## Dedication

This publication is dedicated to people who lost their lives in the COVID-19 pandemic, all the corona warriors. PB dedicates the article in the memory of his father (1958–2021).

## Conflict of interest

The authors declare that the research was conducted in the absence of any commercial or financial relationships that could be construed as a potential conflict of interest.

## Publisher's note

All claims expressed in this article are solely those of the authors and do not necessarily represent those of their affiliated organizations, or those of the publisher, the editors and the reviewers. Any product that may be evaluated in this article, or claim that may be made by its manufacturer, is not guaranteed or endorsed by the publisher.
